# A Novel Classification Method: Neighborhood-Based Positive Unlabeled Learning Using Decision Tree (NPULUD)

**DOI:** 10.3390/e26050403

**Published:** 2024-05-04

**Authors:** Bita Ghasemkhani, Kadriye Filiz Balbal, Kokten Ulas Birant, Derya Birant

**Affiliations:** 1 Graduate School of Natural and Applied Sciences, Dokuz Eylul University, Izmir 35390, Turkey; bita.ghasemkhani@ogr.deu.edu.tr; 2 Department of Computer Science, Dokuz Eylul University, Izmir 35390, Turkey; kadriyefiliz.balbal@deu.edu.tr; 3 Information Technologies Research and Application Center (DEBTAM), Dokuz Eylul University, Izmir 35390, Turkey; ulas@cs.deu.edu.tr; 4 Department of Computer Engineering, Dokuz Eylul University, Izmir 35390, Turkey

**Keywords:** artificial intelligence, machine learning, classification, positive unlabeled learning, decision tree, entropy measure, k-nearest neighbors, supervised learning

## Abstract

In a standard binary supervised classification task, the existence of both negative and positive samples in the training dataset are required to construct a classification model. However, this condition is not met in certain applications where only one class of samples is obtainable. To overcome this problem, a different classification method, which learns from positive and unlabeled (PU) data, must be incorporated. In this study, a novel method is presented: neighborhood-based positive unlabeled learning using decision tree (NPULUD). First, NPULUD uses the nearest neighborhood approach for the PU strategy and then employs a decision tree algorithm for the classification task by utilizing the entropy measure. Entropy played a pivotal role in assessing the level of uncertainty in the training dataset, as a decision tree was developed with the purpose of classification. Through experiments, we validated our method over 24 real-world datasets. The proposed method attained an average accuracy of 87.24%, while the traditional supervised learning approach obtained an average accuracy of 83.99% on the datasets. Additionally, it is also demonstrated that our method obtained a statistically notable enhancement (7.74%), with respect to state-of-the-art peers, on average.

## 1. Introduction

In the domain of artificial intelligence (AI), machine learning (ML) emerges as a dynamic and rapidly evolving field. It empowers technological platforms to glean insights from historical data and predict outcomes or make determinations autonomously based on past observations. ML enables systems to spontaneously identify patterns within large datasets, thereby driving innovation and efficiency across diverse fields. Supervised learning, a fundamental branch of machine learning, lies at the heart of many intelligent systems. It offers great advantages in many different domains, including robotics [1], geology [2], security [3], health [4,5], land cover [6], remote sensing [7], industrial applications [8], and environmental monitoring [9]. Supervised learning allows automated machinery to learn from labeled data, where inputs are paired with corresponding outputs.

In supervised learning, labeling data is a crucial yet intricate process that involves several challenges. Firstly, it requires deep domain expertise to accurately interpret the data, a resource that may not always be readily accessible. Moreover, the task of labeling might be time-intensive, tedious, and labor-demanding, particularly when handling large datasets or subtle classifications that require meticulous attention to detail. Implementing robust quality control measures and providing clear guidelines to annotators through inter-rater reliability assessments and consensus-building methods are essential to maintain the integrity of the labeled dataset. To overcome these labeling challenges, our study focuses on the design of an efficient ML model, while dealing with both labeled and unlabeled data, instead of fully-annotated data.

In traditional binary classification, both negative and positive labeled instances are typically required in the training set [10]. This necessitates the presence of data representing both the absence and presence of the event of interest. However, there are scenarios where only positive samples are available, posing challenges for standard classification methods. For example, consider a situation where researchers are studying the presence of a particular disease. They may only have access to data from patients who have been positively diagnosed with the disease. Unfortunately, data from individuals who have not been diagnosed yet is unavailable. This limitation arises because “*not being diagnosed*” differs from “*not having the disease*” [11]. In some situations, data can be collected only about patients diagnosed positively. For example, patients with calluses on their skin come to the hospital while people who do not have calluses do not come to the callus clinic. Here, data collection efforts focus on specialized clinics or research studies dedicated to specific medical conditions, where participants may already have been diagnosed with the condition of interest. Additionally, clinical trials recruit participants who have already been diagnosed with the condition being studied, leading to data predominantly featuring individuals with positive diagnoses. In each of these scenarios, the data collection process is influenced by factors such as the nature of the disease or condition being studied, the objectives of the research, and the availability of participants for recruitment.

Some kinds of other scenarios are also formed into a positive unlabeled learning task. For instance, in fraud detection, the public security department mostly provides a list of illegal accounts as positive data. Nevertheless, when it comes to any account outside the list, it is not yet certain whether it is trustable or not; considering them as negative may mislead the system. Due to the uncertain situation, the machine learning model can be trained on positive and unlabeled data [12]. Similarly, in the field of e-commerce, the favorite products of a user are known from his/her shopping cart; however, it is uncertain when it comes to discerning the products that a user does not like.

The absence of negative class samples poses a challenge to data scientists since it does not allow the direct application of traditional classification methods. Furthermore, the presence of unlabeled instances also requires additional effort since the standard classification algorithms, such as support vector machines, are designed for learning from positive–negative data. On the other hand, the presence of unlabeled instances or ambiguous class boundaries can produce positive effects on entropy, further improving classification performance. Furthermore, the automated or semi-automated data labeling process [13], coupled with human oversight, can help to streamline the labeling process, as well as construct effective machine learning models for the classification task [14]. Consequently, innovative approaches have to be developed under the broader category of machine learning, aiming to extract meaningful insights from datasets containing positive and unlabeled instances for binary classification tasks [15,16].

Positive unlabeled learning (PUL) is the process of building a classifier based on a positively labeled and unlabeled dataset when negatively-labeled samples are absent. Its primary emphasis lies in binary classification, particularly in applications related to information retrieval, outlier detection, and novelty detection. Furthermore, its utility extends beyond these purposes, encompassing applications in time series analysis as well [17]. PUL aligns with the ongoing endeavor to devise learning techniques capable of operating with incomplete supervision, including one-class classification [18] and semi-supervised learning [19]. However, PUL sets itself apart from one-class classification methods by directly incorporating unlabeled data into learning tasks. It bears a resemblance to the latter by customizing the traditional semi-supervised approach, which commonly involves labeled examples for all classes. Additionally, to facilitate learning with unlabeled and positive data in the PUL process, it is essential to regard certain assumptions, namely, the mechanism of labeling and the distribution of classes in the dataset [20].

In this investigation, our attention is centered on the intricacies of PU learning and proposing a novel AI method, neighborhood-based positive unlabeled learning using decision tree (NPULUD), aimed at addressing the challenge posed by unlabeled data when only positive labels are available for binary classification. By incorporating the principles of k-nearest neighbors (KNN) and leveraging the strengths of the decision tree, NPULUD intends to boost the accuracy and reliability of classification models in real-world applications as evaluated across a diverse array of datasets. The decision tree algorithm, known for its simplicity and interpretability, is a popular choice for tackling classification tasks, while the utilization of KNN allows NPULUD to capture the local structure of the data and make informed decisions based on neighboring instances. Similarly, the KNN algorithm is renowned for its simplicity and effectiveness in classification, often performing well in practice due to its intuitive nature and ability to adapt to complex data distributions. Additionally, entropy serves as the fundamental measure utilized in the NPULUD method through the process of constructing a decision tree.

The main contributions of this study that distinguish it from various positive unlabeled learning-based methods are as follows:

(i) A novel method, entitled neighborhood-based positive unlabeled learning using decision tree (NPULUD), was unveiled for the first time in this study.
(ii) The presented method learns from datasets containing only positive and unlabeled samples, without the presence of negative samples, for binary classification tasks based on the PU strategy of the nearest neighborhood and decision tree classifier.
(iii) The NPULUD method was evaluated based on 24 real-world datasets from various domains and achieved a high accuracy of 87.24%, compared to the conventional supervised learning method, which had an average accuracy of 83.99%.
(iv) Our method achieved a statistically significant improvement (7.74%), in comparison with its state-of-the-art counterparts, on average.
(v) The presented method consistently outperformed the traditional decision tree (DT) method in precision, recall, and F-measure metrics with average values of 0.8572, 0.8724, and 0.8625, respectively. This underscores its reliability and robustness across diverse datasets.
(vi) Rigorous statistical analysis, including the Wilcoxon test with a *p*-value of 0.0004693, affirmed the superiority of NPULUD over the DT method, reinforcing its effectiveness.

In this exploration, entropy is used to assess the uniformity of the data during the construction of the decision tree. Entropy measure is a key concept used in various fields to quantify uncertainty or randomness within complex data or systems [21]. In the context of decision tree classification, entropy stands as a crucial metric for evaluating the impurity of data at different nodes of the tree. The most frequently utilized type of entropy in decision tree classification is the Shannon entropy [22]. Shannon entropy measures the uncertainty in a dataset by calculating the distribution of class labels. Other types of entropy measures, such as the Kullback–Leibler divergence [23], possess applications tailored to various fields of study. However, in the context of decision tree classification, Shannon entropy stands out as the primary measure employed to guide the splitting of nodes and optimize the classification process.

The subsequent sections of this paper unfold as follows: Section 2 offers a succinct review of related works. In Section 3, we delve into the materials and methods utilized, followed by Section 4, which provides experimental studies conducted. Lastly, in Section 5, we present the conclusions drawn from our findings and outline avenues for future works on the proposed method.

## 2. Related Works

PUL has found applications across various domains, including bioinformatics [24,25,26], network link prediction [27], text classification [28], transportation [29], and image processing [30,31,32,33,34]. In the field of bioinformatics [24], the authors systematically reviewed various biological problems in 29 different bioinformatics applications based on PUL. The study states that the main problem in the field of bioinformatics is the loss of well-labeled negative data. According to [24], this shortcoming presents difficulties in enhancing traditional machine learning applications. In this case, using a PUL method can provide high performance for critical problems. In [25], the authors proposed the positive sample-only learning (PSoL) approach that does not require negative training data. When they compared their results with the five other different studies, they achieved higher prediction performance (80%) with the PSoL approach. In [26], Yang et al. proposed an ensemble-based positive unlabeled (EPU) learning approach for different gene identifications through combining pseudo-biological datasets. This change achieved significantly better results. They stated that by minimizing potential errors arising from data in the field of bioinformatics with the EPU method, more accurate and robust predictions can be obtained compared to traditional machine learning approaches.

In the field of network link prediction [27], Gan et al. conducted experiments on three different network datasets with three PUL techniques to improve prediction performance. They obtained promising results improving classification performance with PUL approaches consisting of Standard-PU (positive unlabeled learning utilizing conventional classifier prediction), Bagging-PU (positive unlabeled learning employing bagging methodology), and TwoStep-PU (positive unlabeled learning applying trustworthy negative sampling) techniques. In [28], successful results were achieved in the field of text classification by utilizing unlabeled and positive instances. Liu et al. performed their analysis with a limited set of labeled positive data, primarily comprising text data, alongside an extensive collection of unlabeled data. In their study, unlabeled data helped the algorithm learn better. In the transportation field [29], Sevetlidis et al. suggested a PUL method for accident classification in black spots to improve road safety. When they compared their results with those of supervised learning, they obtained better results in terms of accuracy, recall, precision, F-measure, and area under the curve (AUC) values.

In the field of image processing [30], Wang et al. used PUL, isolation forest, and one-class support vector machine (OCSVM) algorithms for single-class classification. They stated that PUL, which stands out for its advantages of ease of parameter adjustment and controllable training time, also demonstrated the highest performance. In another study [31], Wang et al. used a PUL method to accurately identify invasive plants using satellite images. In their study, carried out with three different datasets and utilizing decision trees as the basic classifier, they were able to identify the invasive plant Pedicularis with an accuracy exceeding 0.70 in all datasets using the PUL method. In [32], Li et al. conducted a study in geospatial observation classification aimed at classifying a particular land use type. They utilized PUL, Gauss field, OCSVM, and biased support vector machine (SVM) methods for this purpose. According to the experimental results of their study, in which high spatial resolution photographs were classified, the PUL algorithm demonstrated higher performance compared to other single-class classification methods.

In another study [33], urban areas in the United States were mapped using PUL algorithms and moderate resolution imaging spectroradiometer (MODIS) datasets. In this study, the total correctness of the city map obtained by the PUL method was found to be 92.91% (Kappa = 0.85). The prediction results obtained with PUL were similar to the National Land Cover Database (NLCD) urban map in terms of the urban areas of medium and small cities. Similarly, Desloires et al. [34] implemented grain and forest mapping based on satellite images in a region in the southwest of France. They proposed positive and unlabeled learning for the satellite image time series (PUL-SITS) method, which consists of two stages. Their study was conducted with two different scenarios aimed at classifying grain and forest lands from satellite images and proving the success of the proposed method.

The literature review summary is represented in Table 1. As base classifiers, various machine learning methods are employed in prior studies, incorporating SVM [27,29,30,32], neural networks [35], KNN [29,36], logistic regression (LR) [27], modified logistic regression (MLR) [37], decision tree (DT) [27], and naive Bayes (NB) [27]. Additionally, studies have utilized ensemble learning [34], particularly random forest (RF) [27,29,34]. Furthermore, the deep learning approach has also been used in some of the previous studies [29,38]. Some studies [27,29,35] preferred the k-fold cross-validation technique while others [34,36] utilized the train-test split procedure. While most studies [27,29,34,36] evaluated the results using the accuracy and F-measure metrics, some of them [29] also used precision and recall metrics, and others [27,34,35] utilized different indicators like specificity, sensitivity, kappa, and the area under the precision-recall curve (AUPR).

It is noteworthy to mention that several studies have also explored the use of entropy measures in their methodologies. For instance, Zahoor et al. [39] incorporated entropy-based feature extraction to enhance the performance of classification via a modified entropy whale optimization algorithm. Howedi et al. [40] utilized entropy as a measure for detecting anomalies in daily activities during visitor presence and analyzed the collected information through passive infrared and door entry sensors in a home environment. Hasan et al. [41] introduced a novel method to categorize MRI brain scans utilizing deep learning and quantum entropy characteristics with the aim of augmenting the precision of brain tumor identification in the early stages.

Different from the previous studies, in this work, the NPULUD method was introduced as a novel approach that surpasses existing methods, advancing the field of positive unlabeled learning. It addresses challenges and extends applications in scenarios with limited labeled data.

## 3. Materials and Methods

### 3.1. Proposed Method

The current paper proposes a novel classification method—entitled neighborhood-based positive unlabeled learning using decision tree (NPULUD)—to train on positive and unlabeled (PU) data. In this method, nearest neighborhood and decision tree approaches are utilized as the PU strategy and base classifier, respectively.

**Figure 1:**
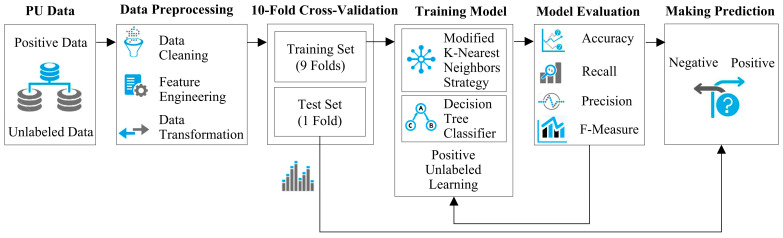
A general overview of the presented method.

Figure 1 shows an outline of the suggested method, in which a positive unlabeled dataset is ready to be processed for cleaning data, engineering features, transforming data, and other alternative preparation phases if required. The dataset undergoes 10-fold cross-validation, where it is randomly segmented into ten folds. One fold is reserved for testing while the remaining nine folds are used for training. This process is iterated 10 times to evaluate the classifier’s performance and compute the average classification rate. In the training step, the concept of PUL, based on the nearest neighbors strategy and decision tree classifier, is integrated to construct the core of the NPULUD method. Afterward, the evaluation will be conducted using different criteria like accuracy, recall, precision, and F-measure. This assessment will involve using the test set to make predictions. The predictive model will classify instances into positive and negative categories.

When performing the PU task, we used a revised KNN approach. In particular, the main difference from the traditional KNN approach is that it requires all neighbors to be positive instances for a final class decision, instead of most neighbors. In other words, our method considers a unanimous voting strategy, instead of the majority voting scheme in KNN.

For a given input *x*, the KNN algorithm identifies *k* samples within the training subset that are nearest to *x* with a predefined distance measure and makes a prediction by majority vote from the classes of the *k* samples. Even though it is a simple and useful method, it can make wrong predictions when the number of votes are close to each other (i.e., 2 neighbors positive and 3 neighbors negative for *k* = 5). For example, with five neighbors or *k* = 5, the estimated probability takes the value 60% when 3 neighbors belong to the class. To overcome this drawback, we propose a different method that identifies *strong positive instances* with 100% probability.

**Definition 1.** **(Strong Positive Instance).** *For a new test point x ∈ T, its k-nearest neighbors* $N_{k}(x)$ *are found by calculating the distances between x and the instances in the dataset D. An instance x is called a strong positive instance if every sample x_i_ in* $N_{k}(x)$ *belongs to a positive class (P_L_), such as in the following Equation (1):*

(1) $$k=sign\left(\sum_{x_{i}\in N_{k}(x)}y_{i}\right)$$

The binary classification aims to build a classifier that has the ability to differentiate the input as positive or negative, based on its features. To construct a classifier, the algorithm uses a set of training samples. In dataset *D*, each training sample is a pair (*x*, *y*), where *x* is a vector in a feature space and *y* is the corresponding class value. Conventional supervised learning algorithms work on the training subset that is presumed to be completely labeled. This means that class values (positive or negative) for every training sample are known. Table 2 illustrates an instance of a meticulously labeled training subset for supervised learning.

The aim of PUL is the same as standard classification: build a classifier that can correctly assign the appropriate class label (positive and negative) to a given input. However, in the learning stage, only a portion of the positive samples in the training subset are labeled, and the negative samples are not addressed. The PU dataset can be presented as *D* = *P_L_* + *U*, where *P_L_* consists of pairs (*x*,*y*) while *U* includes tuples with only *x* (a vector of features). Table 3 illustrates an example of a positive and unlabeled training dataset, which consists of instances labeled as just positive and unlabeled ones.

**Figure 2:**
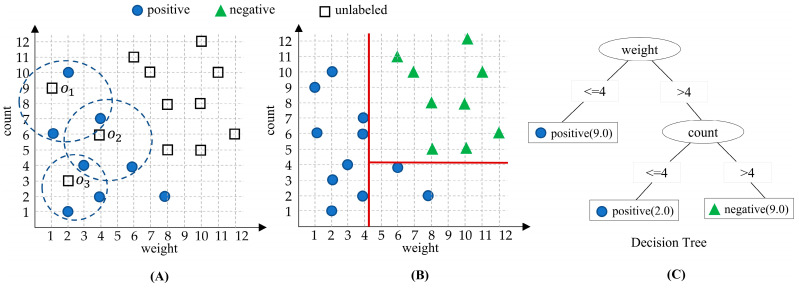
An illustration of the proposed method by an example.

Here, we aim to describe the proposed NPULUD method via a simple example, designed specifically to learn from positive and unlabeled instances (Table 3) to ultimately classify positive and negative ones, as presented in Table 2. The training set comprises 20 instances characterized by numerical features such as weight and count features, along with a categorical target class. The method begins by applying the modified KNN algorithm with the Euclidean distance metric to identify the nearest neighbors for each unlabeled instance in the dataset, shown in Figure 2A. With a predefined number of neighbors *k* = 3, the distances between each unlabeled instance and its nearest positive instances are calculated. If all neighbors belong to the positive class, the current object (i.e., $o_{1}$) is assigned to the positive category, alternatively to the negative one. The labeling process iteratively continues for each unlabeled instance, as shown in Figure 2B.

Following the labeling of unlabeled instances using the modified KNN approach, we used a decision tree classification algorithm to build a classifier. The algorithm learns from the labeled instances. By leveraging the decision tree’s ability to capture complex decision boundaries and interactions among features, it is supposed to achieve enhanced classification performance compared to the initial labeling step. As shown in Figure 2C, the decision tree splits instances based on their weight attribute. If the weight is greater than 4, the tree further splits based on the count attribute. Similarly, if the count exceeds 4, the instance is identified as negative, and otherwise, as positive. If the weight is 4 or less, the tree directly classifies based on the number of positive instances. Also, if the count is less than 4, the instance is regarded as positive. This decision tree offers a straightforward classification process using weight and count features, facilitating the effective classification of instances in PU data.

### 3.2. Formal Description

In the context of PU learning, datasets are commonly delineated using set notations, wherein a dataset *D* consists of instances from positive labeled ${P_{L}=\{\left(x_{i},y_{i}\right)\}}_{i=1}^{n_{p}}$ and unlabeled *U*${=\{\left(x_{i}\right)\}}_{i=1}^{n_{u}}$ sets, expressed as *D* = *P_L_* + *U*. Similarly, set *P* incorporates instances from positive labeled (*P_L_*) and positive unlabeled (*P_UL_*) subsets, represented as *P* = *P_L_* + *P_UL_*. The objective of PU learning involves training a binary classifier exclusively using instances from sets *P* and *U*. Specifically, learning from PU data offers a modification of the classical binary classification model. In this modification, the training set exclusively comprises positive and unlabeled data points. First, the algorithm aims to identify positive unlabeled (*P_UL_*) and negative unlabeled (*N_UL_*) subsets, denoted as *U* = *P_UL_* + *N_UL_*. After that, it employs a standard classification task.

Employing these formal notations, conventional classification tasks are viewed as establishing a decision border delineating sets *P* and *N* to accurately categorize instances into positive and negative classes based on identified features, thus outlining a clear separation between the two sets. Generalizing to new instances is vital in practical applications in which the model needs to make predictions on previously unseen data. This structured approach aids in the analysis and advancement of PU learning methodologies. Understanding the relationship between traditional classification and PU learning is essential for developing robust methods. Exploring these connections can improve PU learning algorithms, emphasizing the importance of the PU strategy and its base classifier model in effectively distinguishing between positive and negative instances.

Algorithm 1 represents the pseudocode of the suggested NPULUD approach. The initial step begins with the inputs, namely, *U* representing the unlabeled set consisting of *n_u_* instances; *P_L_* denoting the positive set comprising *n_p_* instances, where *y_i_* represents the class label associated with *x_i_* from the *P_L_* set; *D* combining *P_L_* and *U* sets to produce the positive unlabeled dataset; *k* indicating the number of neighbors to consider in the nearest neighbors search; and *T*, the test set containing unseen instances to be predicted. The output *C* contains the predicted class labels for all instances in the test set *T*. Then, the NPULUD algorithm initializes an empty set *P_UL_* to store potential positive unlabeled instances.

The algorithm iterates over each unlabeled instance *x* in the input set *U*. For each *x*, it computes the *k* nearest neighbors of *x* in the combined dataset *D* using the nearest neighbors function assigned as *N_k_*. The number of positive objects of *N_k_* is counted for each instance *x*, and if all *k* neighbors are positive, signifying a dense region of positive instances, the algorithm adds the instance to the set *P_UL_*; otherwise, the current object is assigned as negative. In this way, the algorithm iteratively identifies the potential positive unlabeled (*P_UL_*) and negative unlabeled (*N_UL_*) instances. After that, the algorithm constructs a decision tree model *M* using the positive labeled instances *P_L_*, the recognized positive unlabeled instances *P_UL_*, and any potential negative unlabeled instances *N_UL_*. Finally, for every instance in the test subset *T*, the algorithm predicts its class label using the decision tree model *M*, and aggregates these predicted labels into the output *C*, which contains the predicted class labels for all instances in the test set *T*.
**Algorithm 1:** Neighborhood-based Positive Unlabeled Learning Using Decision Tree    (NPULUD)**Inputs:**
  *U*: unlabeled set $U{=\{\left(x_{i}\right)\}}_{i=1}^{n_{u}}$
  P_L_: positive set ${P_{L}=\{\left(x_{i},y_{i}\right)\}}_{i=1}^{n_{p}}$
  D = P_L_ + *U*
  *k =* number of neighbors
  *T*: test set that will be predicted
**Outputs:**
  *C*: predicted class labels**Begin:**
  P_UL_ = Ø
  **foreach**
*x*
**in**
*U*
     $N_{K}\left(x\right)$ = *NearestNeighbors*(*D, x, k*)
     *count* = 0
     **foreach** *object* **in** $N_{K}\left(x\right)$
     if *object* is positive
      *count++*
     **end foreach**
     **if (**
*k* = *count*
**)**
     P_UL_.Add(*object*)
     **else**
     N_UL_.Add(*object*)
     **end if**
  **end foreach**
  *M = DecisionTree(*P_L +_ P_UL_ + N_UL_*)*
  **foreach**
*x*
**in** T
     *c* = M(*x)* // prediction
     *C* = *C* U *c*
  **end foreach** **End**

The time complexity of the NPULUD algorithm is O (T*k + *L*(*n*)), where *T* represents the time required in the nearest-neighborhood process, *k* signifies the number of neighbors, and *L(n)* denotes the time needed for executing the DT approach on *n* instances.

To this research, entropy pertains to the measure of evaluating the uniformity or heterogeneity within the dataset during the construction of decision trees. At every node of the tree, the decision on how to split the data is derived from the concept of normalized information gain ratio, which is essentially the difference in entropy. A substantial entropy score indicates that the distribution of samples across different classes is relatively balanced, on the other hand, an insignificant entropy score indicates that one class dominates the dataset. The goal of the algorithm is to decrease entropy, as lower entropy implies higher certainty in classifying instances. The entropy of a training dataset *D* is calculated based on the target attribute using Equation (2), as follows:

(2) $$Entropy\left(D\right)=\sum_{i=1}^{m}R_{i}\log_{2}R_{i}$$

Here, *R_i_* represents the proportion of samples associated with class *i*, and *m* stands for the total count of classes.

The total of proportion values should be 1, as shown in Equation (3):

(3) $$\sum_{i=1}^{m}R_{i}=1$$

If a feature $F_{i}$ is generated with *v* values, this leads to splitting the dataset *D* into *v* subsets, such as $D_{1},D_{2}{\ldots ,D}_{v}$. For the feature $F_{i}$, the expected entropy is calculated as given in Equation (4):

(4) $${Entropy}_{F_{i}}\left(D\right)=-\sum_{j=1}^{v}\frac{\left|D_{j}\right|}{D}*Entropy(D_{j})$$

Information gain for choosing feature $F_{i}$ to partition the data is given in Equation (5):

(5) $$Entropy\left(D,F_{i}\right)=Entropy(D)-{Entropy}_{F_{i}}\left(D\right)$$

The feature with the highest gain was selected to split the current tree node.

## 4. Experimental Studies

The primary objective of this study is to appropriately classify the positive and negative classes in a positive unlabeled dataset. To reach this goal, a novel method entitled neighborhood-based positive unlabeled learning using decision tree (NPULUD) is proposed with the PU strategy of nearest neighborhoods and the classifier of a decision tree. The C4.5 decision tree (DT) algorithm is chosen as the primary classifier on account of its effectiveness. This algorithm employs entropy, a potent measure for guiding the partitioning of data within tree nodes. The competence of the NPULUD method was approved over 24 positive unlabeled datasets in various fields. Our method is implemented using the C# programming language with integration of the Weka library [42]. After testing different parameter values, the number of neighbors (*k*) is configured to 3 in the experiments. During experimentation, we employed the 10-fold cross-validation approach to train and evaluate the classifier. In the current method, the dataset is randomly segmented into 10 folds. One of the folds is set aside as the testing set, while the other nine folds are utilized as the training set. This process is iterated 10 times, and the average classification rate is computed. Furthermore, to measure the performance of the presented method, we engaged various evaluation metrics, including accuracy (ACC), precision (PR), recall (R), and F-measure (FM), as defined in Equations (6)–(9), respectively:

(6) $$ACC=\frac{TN+TP}{TN+TP+FN+FP}$$

(7) $$PR=\frac{TP}{FP+TP}$$

(8) $$R=\frac{TP}{FN+TP}$$

(9) $$FM=\frac{2TP}{2TP+FN+FP}$$

where, true positive (TP) signifies instances correctly identified as positive by the classifier, indicating its accuracy in recognizing the presence of the condition or event. On the other hand, true negative (TN) represents instances correctly identified as negative, demonstrating the ability of the classifier to accurately discern the absence of the condition or event. False positive (FP) refers to instances erroneously classified as positive, while false negative (FN) indicates instances falsely categorized as negative. These metrics are pivotal in evaluating the effectiveness of classification models, offering insights into prediction accuracy and the capacity of models to distinguish between classes.

### 4.1. Dataset Description

This research uses 24 various real-world datasets, which are open to the public and available in the UCI Machine Learning Repository [43] to present the functionalities of the suggested NPULUD method. These datasets encompass various instances, spanning from 100 to 48,842, with attributes varying from 3 to 60. The datasets come from different domains, including health, business, life, environment, biology, physics, chemistry, social science, and computer science. These datasets contain diverse types of values, comprising categorical, numerical, and mix-type. It is obvious that numerous studies in the literature utilize these datasets extensively.

Before the training phase, real-world datasets are undertaken through a series of data preparation processes, including data cleaning (to address missing values, outliers, and inconsistencies), data discretization (to convert numerical variables into categorical representations), data normalization (to standardize the dataset to ensure uniformity and to scale values to a common range), data transformation (to encode categorical variables), and feature engineering (to extract relevant features). The benchmark datasets that were used in this study are almost ready-to-use for comparison in research studies. For the present study, the id attributes were removed from the datasets since they do not provide any real insight or contribution during the classification task.

Table 4 summarizes the properties of the datasets, such as the number of data instances (#Instances), the number of features (#Features), the subject area, the number of views (#Views), and other relevant attributes, facilitating a better understanding of them. Notably, for each dataset, 5% of the instances are specified as unlabeled instances. Table 4 also shows the distribution of positive labeled and unlabeled data across the datasets.

### 4.2. Results

The assessment of accuracy metrics between the proposed neighborhood-based positive unlabeled learning using decision (NPULUD) method and the traditional supervised learning-based decision tree (DT) is showcased over 24 datasets in Table 5. The average accuracy across all datasets for NPULUD stands at 87.24%, notably higher than DT’s average of 83.99%. In analyzing results, it is evident that NPULUD consistently exhibits superior performance over DT in the majority of cases (20 out of 24). This overall superiority of NPULUD underscores its potential as a robust alternative to traditional supervised learning approaches, particularly in scenarios where labeled data may be scarce or expensive to acquire. For instance, in the “fertility-diagnosis” dataset, NPULUD achieved 94.00% accuracy compared to DT’s 85.00%. Similarly, in the “heart-statlog” dataset, NPULUD attained 84.44% accuracy compared to DT’s 76.67%, and in the “thoracic-surgery” dataset, NPULUD gained 84.47% accuracy compared to DT’s 92.13%. These results emphasize the reliable outperformance of the NPULUD method over traditional supervised learning methods across various datasets, indicating its capability for improving predictive accuracy in a range of applications.

**Figure 3:**
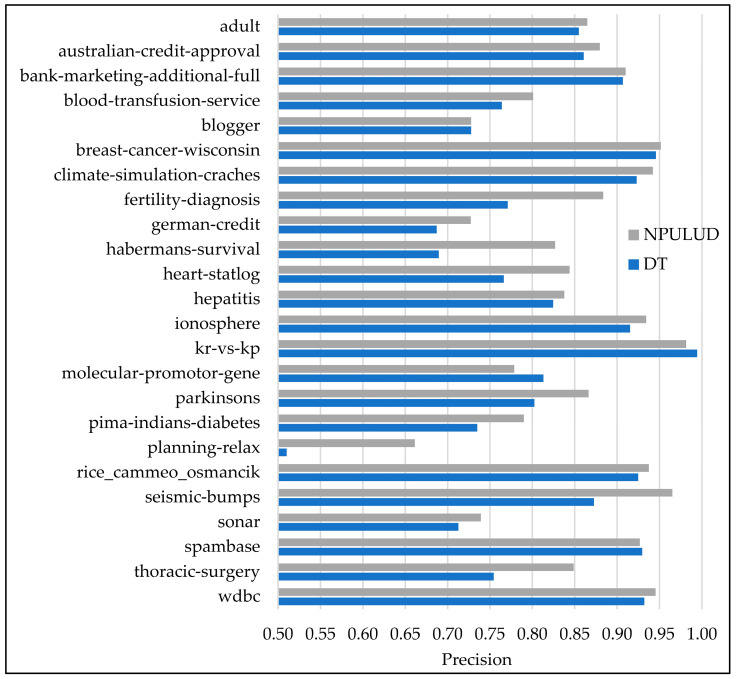
A comparison of the supervised learning and NPULUD method in precision.

**Figure 4:**
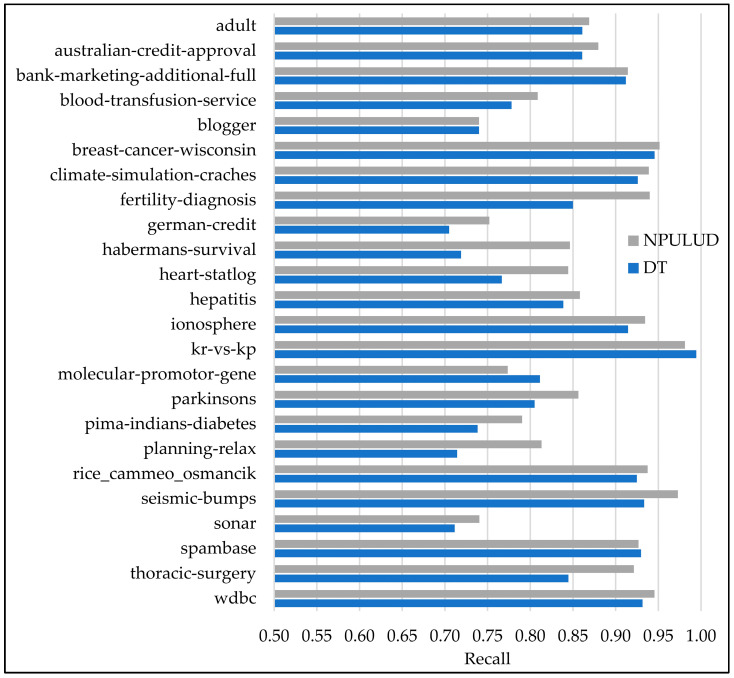
A comparison of the supervised learning and NPULUD method in recall.

**Figure 5:**
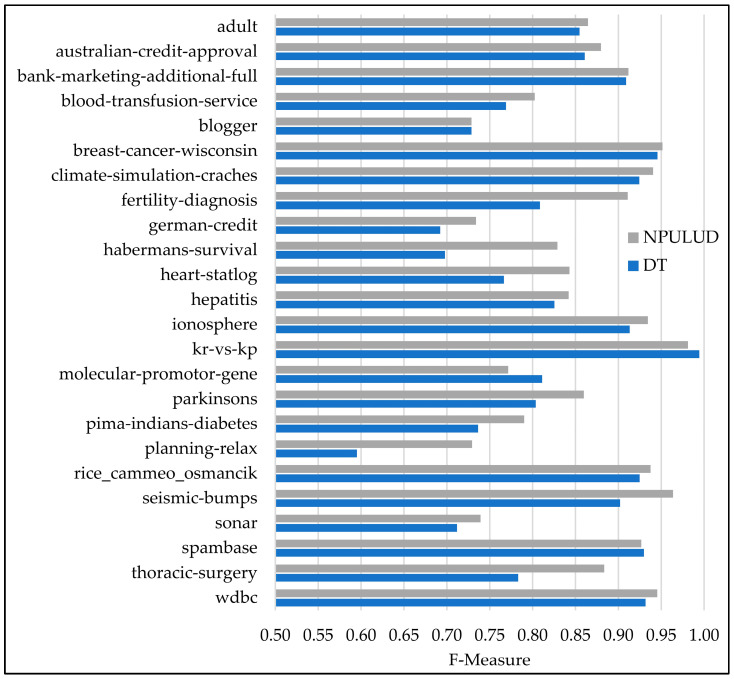
A comparison of the supervised learning and NPULUD method in F-measure.

In Table 5, the comparison between the DT and NPULUD methods based on accuracy metric has been previously detailed. Here, we focus on results based on precision, recall, and F-measure metrics represented in Figure 3, Figure 4 and Figure 5, respectively. The NPULUD method consistently surpasses DT across all 24 datasets with averages of 0.8572, 0.8724, and 0.8625 in precision, recall, and F-measure metrics, respectively, in comparison with DT method with averages of 0.8175, 0.8399, and 0.8259 in precision, recall, and F-measure metrics, respectively. Moreover, NPULUD outperforms DT in all the mentioned metrics in 20 out of 24 datasets when comparing results from each dataset. For instance, in the “seismic-bumps” dataset, NPULUD achieves a high precision of 0.9652, outperforming DT, which achieves 0.8727. Conversely, in the “planning-relax” dataset, NPULUD records the lowest precision of 0.6613, still surpassing DT’s precision of 0.5102. Similarly, NPULUD exhibits a high precision, recall, and F-measure in the “habermans-survival” dataset at 0.8269, 0.8464, and 0.8290, respectively, while DT obtains 0.6895, 0.7190, and 0.6978, respectively, demonstrating steadily superior performance of NPULUD method across all mentioned metrics with a persistent trend. The given examples highlight the robustness of NPULUD over various datasets, indicating its superiority in capturing evaluation aspects of the classification task compared to DT. This shift in focus from accuracy to precision, recall, and F-measure metrics provides a more sophisticated evaluation of algorithmic performance, enriching an understanding of the NPULUD efficiency in real-world classification tasks.

Furthermore, drawing from the outcomes of the Wilcoxon test to compare the accuracies of supervised learning (SL) and positive unlabeled (PU) learning across 24 datasets, a *p*-value of 0.0004693 is obtained at a significance level of 0.05. Here, the Wilcoxon test is a non-parametric statistical analysis that is employed to make comparisons between paired groups when data does not meet parametric assumptions. Additionally, the *p*-value represents the probability of obtaining test results as extreme as the observed ones if the null hypothesis were true. Thus, with the obtained *p*-value substantially less than the significance level as strong evidence, the null hypothesis of no disparity between the accuracies of SL and PU learning is rejected. This reveals a statistically substantial difference in accuracy between the learning methods across the datasets. It is concluded that the PU learning method consistently outperforms the SL learning method, suggesting that the choice between positive unlabeled learning and supervised learning expressively impacts accuracy outcomes in the context of the analyzed datasets.

We investigated the effectiveness of the NPULUD method across varying proportions of positively labeled instances and unlabeled instances (P/U) in the training set. To investigate this, we conducted extensive testing on all 24 datasets, manipulating the P/U ratios at 95/5%, 90/10%, 85/15%, and 80/20%. The results of these experiments are presented in Table 6, where each dataset is evaluated under different P/U ratios, facilitating clarity and comparison. The findings revealed that the NPULUD method showed good and robust performance across a spectrum of P/U ratios. It achieved an average accuracy of 87.24%, 86.34%, 86.18%, and 86.07% for unlabeled data ratios of 5%, 10%, 15%, and 20%, respectively, demonstrating its robustness across various ratios. Notably, the results indicated that the method obtained the best performance at a 95/5% ratio, albeit with minor differences. For example, for the “adult” dataset, the method demonstrated an accuracy of 86.90% for a 5% unlabeled data ratio, 86.71% for a 10% unlabeled data ratio, 86.51% for a 15% unlabeled data ratio, and 86.55% for a 20% unlabeled data ratio. In each case, the method performed well, and the accuracy values are acceptable.

### 4.3. Comparison with Respect to State-of-the-Art Method

Within this section, the presented NPULUD method was contrasted with the other positive unlabeled learning algorithms—AdaSingle-SVM, AdaEnsemble-SVM, AdaSingle-KNN, and AdaEnsemble-KNN [44]—regarding F-measure. Table 7 indicates the results that were directly outlined in the state-of-the-art study on the same datasets, including breast-cancer-wisconsin, ionosphere, pima-indians-diabetes, sonar, and wdbc. According to the results, the NPULUD outperformed the previous PUL algorithms across all datasets, except the breast-cancer-wisconsin dataset. For instance, NPULUD (79.04%) indicated its superiority over AdaSingle-SVM (65.50%), AdaEnsemble-SVM (66.00%), AdaSingle-KNN (61.10%), and AdaEnsemble-KNN (62.40%) on the pima-indians-diabetes dataset. In particular, the most significant disparity in accuracy between NPULUD and the remaining was noted on the ionosphere dataset, where NPULUD enhanced the performance by over 18% against the AdaSingle-KNN. As shown in the results, the NPULUD method acquired the highest performance (87.24%) on average in comparison with the previous methods. Consequently, the proposed method showcased its superiority over its counterparts with an average of 7.74% improvement.

## 5. Conclusions and Future Work

In this study, we addressed the challenge of constructing classification models when only one class of samples and unlabeled samples are available, a scenario commonly encountered in various real-world applications. To mitigate this limitation, we introduced a new classification method termed neighborhood-based positive unlabeled learning using decision tree (NPULUD). Our approach leverages positive and unlabeled (PU) data by employing the nearest neighborhood approach followed by a decision tree algorithm for classification. Here, entropy is employed as the fundamental measure for generating the decision tree classifier.

Through experiments conducted on 24 real-world datasets, we demonstrated the effectiveness of NPULUD in accuracy metrics, achieving a higher average performance of 87.24% compared to supervised learning (SL) at 83.99%. In addition, other performance evaluation metrics, including precision, recall, and F-measure, are investigated across all 24 datasets, demonstrating that the NPULUD method consistently outperforms SL, with average scores of 0.8572, 0.8724, and 0.8625, compared to DT’s averages of 0.8175, 0.8399, and 0.8259, respectively, in these metrics. Moreover, the Wilcoxon test confirmed a statistically significant difference in accuracy between PU learning and SL methods with a *p*-value of 0.0004693 across the datasets, reaffirming the consistent outperformance of PU learning. Further analysis corroborated the superiority of our method, with a significant improvement of 7.74% accuracy over state-of-the-art competitors. These findings underscore the potential of NPULUD as a robust alternative for classification tasks when labeled data is scarce or unavailable.

The study presents several key contributions that distinguish it from existing positive unlabeled learning-based methods:

(i) Introduction of NPULUD: This study introduces NPULUD, a novel method that has not been previously documented in the literature, and learns from datasets comprising solely positive and unlabeled samples, eliminating the need for negative samples in binary classification tasks.

(ii) Unique learning approach: NPULUD offers a unique learning approach by effectively utilizing only positive and unlabeled data for classification tasks. By employing the nearest neighborhood PU strategy in conjunction with a decision tree classifier, NPULUD demonstrates its capability to derive meaningful insights and accurate predictions from limited data scenarios.

(iii) Evaluation of diverse real-world datasets: The effectiveness of NPULUD is rigorously evaluated across 24 real-world datasets spanning various domains. Remarkably, NPULUD achieves a high accuracy rate of 87.24%, surpassing traditional supervised learning methods which exhibit an average accuracy of 83.99%. This underscores the robustness and versatility of NPULUD across different data environments.

(iv) Statistically significant improvement: NPULUD demonstrates a statistically significant improvement of 7.74% on average when compared to state-of-the-art counterparts. This notable enhancement in performance further validates the efficacy of NPULUD as a superior alternative for positive unlabeled learning tasks.

(v) Superior performance across multiple metrics: In addition to achieving higher accuracy compared to traditional supervised learning methods, NPULUD consistently outperforms in other performance evaluation metrics such as recall, precision, and F-measure, highlighting the comprehensive effectiveness of NPULUD in producing reliable and robust classification results across diverse datasets.

(vi) Statistical validation of method superiority: The statistical analysis conducted in the study, including the Wilcoxon test, provides empirical evidence of the superiority of NPULUD over traditional supervised learning methods with a *p*-value of 0.0004693.

(vii) Contribution to advancing positive unlabeled learning approach: The NPULUD method contributes to the advancement of positive unlabeled learning by introducing a novel method that offers significant performance improvements compared to existing approaches. By addressing key challenges and providing innovative solutions, NPULUD expands the scope and potential applications of positive unlabeled learning methodologies.

While our study has demonstrated promising results for NPULUD, several opportunities for prospective investigations exist to further enhance the applicability of the method. Firstly, NPULUD can be adapted for multi-class PU learning. Furthermore, exploring the integration of NPULUD with sophisticated machine learning techniques, such as deep learning or meta-learning architectures, could unlock new possibilities for the classification task in challenging data environments. Overall, addressing these opportunities for future research will contribute to advancing the field of positive unlabeled learning in real-world applications.

## Figures and Tables

**Table 1:** Literature review summary.

Author(s)	Year	Method	PU Strategy	Base Classifier	Application	Validation	Evaluation Metrics
Sevetlidis et al. [29]	2024	PUL framework	Deep Learning Isolation Forest	Poisson, Gaussian Process, KNN, SVM, RF, XtraTrees, MLP	Black spot accident identification	k-fold cross-validation (k = 5)	Accuracy Recall Precision AUC F-measure
Gan et al. [27]	2022	Standard-PU (RF)	Standard Bagging Two-Step	RF, SVM, LR, DT, NB	Network link prediction	k-fold cross-validation	Accuracy AUPR AUC F-measure
Park [36]	2022	PUL, MPUL	Non-negative Risk Estimator for PU	KNN	Outlier detection in a low dimensional embedding space	train-test split (20–80%) (20 times)	Accuracy F-measure
Yan et al. [35]	2021	EmptyNN	None	Neural Networks	Single-cell RNA sequencing quality control	k-fold cross-validation (k = 10)	Sensitivity Specificity
Desloires et al. [34]	2021	PUL-SITS	PUL-SITS noReg, PUL-SITS reco	RF, Ensemble, OCSVM PUL-SITS	Cereal and forest mapping	train-test split 0.5	Accuracy Sensitivity Specificity Kappa F-measure
Jaskie et al. [37]	2021	MLRf	None	MLR, MLRf, Naïve PU, Oracle, Tiny Supervised Learning	Solar fault detection	unspecified	F-measure
Barcelos et al. [38]	2021	PUL	FCM-GO, FCMFP	Deep learning	Current-based bearing fault diagnosis	unspecified	Accuracy
Proposed		NPULUD	Modified KNN	DT	General purpose	k-fold cross-validation (k = 10)	Accuracy Recall Precision F-measure

**Table 2:** Supervised learning training set example.

ID	Weight	Count	Class
1	1	6	positive
2	1	9	positive
3	2	1	positive
4	2	3	positive
5	2	10	positive
6	3	4	positive
7	4	2	positive
8	4	6	positive
9	4	7	positive
10	6	4	positive
11	6	11	negative
12	7	10	negative
13	8	2	positive
14	8	5	negative
15	8	8	negative
16	10	12	negative
17	10	5	negative
18	10	8	negative
19	11	10	negative
20	12	6	negative

**Table 3:** Positive unlabeled learning training set example.

ID	Weight	Count	Class
1	1	6	positive
2	1	9	unlabeled
3	2	1	positive
4	2	3	unlabeled
5	2	10	positive
6	3	4	positive
7	4	2	positive
8	4	6	unlabeled
9	4	7	positive
10	6	4	positive
11	6	11	unlabeled
12	7	10	unlabeled
13	8	2	positive
14	8	5	unlabeled
15	8	8	unlabeled
16	10	12	unlabeled
17	10	5	unlabeled
18	10	8	unlabeled
19	11	10	unlabeled
20	12	6	unlabeled

**Table 4:** Properties of the datasets.

ID	Dataset Name	#Instances	#Features	Positive Labeled/ Unlabeled Ratio	Subject Area	#Views	Date	Missing Value	Dataset Type	Feature Type
1	adult	48,842	14	46,400/2442	Social Science	362,145	1996	Yes	Multivariate	Categorical, Integer
2	australian-credit approval	690	14	655/35	Business	35,680	N/A	Yes	Multivariate	Categorical, Real, Integer
3	bank-marketing	45,211	16	42,950/2261	Business	194,286	2012	No	Multivariate	Categorical, Integer
4	blogger	100	5	95/5	Computer Science	2854	2013	No	Multivariate	Categorical
5	blood-transfusion service	748	4	711/37	Business	14,491	2008	No	Multivariate	Real
6	breast-cancer- wisconsin (diagnostic)	699	9	664/35	Health and Medicine	104,651	1992	Yes	Multivariate	Integer
7	climate-simulation-craches	540	18	513/27	Climate and Environment	5636	2013	No	Multivariate	Real
8	fertility-diagnosis	100	10	95/5	Health and Medicine	16,444	2013	No	Multivariate	Real
9	german-credit	1000	20	950/50	Social Science	118,674	1994	No	Multivariate	Categorical, Integer
10	habermans-survival	306	3	291/15	Health and Medicine	23,248	1999	No	Multivariate	Integer
11	heart-statlog	270	13	256/14	Health and Medicine	33,711	N/A	No	Multivariate	Categorical, Real
12	hepatitis	155	19	147/8	Health and Medicine	50,330	1988	Yes	Multivariate	Categorical, Real, Integer
13	ionosphere	351	34	333/18	Physics and Chemistry	52,935	1988	No	Multivariate	Real, Integer
14	kr-vs-kp (king-rook vs. king-pawn)	3196	37	3036/160	Games	20,686	1989	No	Multivariate	Categorical
15	molecular-promotor-gene (molecular biology promoter gene sequences)	106	57	101/5	Biology	25,484	1990	No	Sequential, Domain- Theory	Categorical
16	parkinsons (with replicated acoustic features)	240	9	228/12	Health and Medicine	4386	2019	No	Multivariate	Categorical, Real, Integer
17	pima-indians- diabetes	768	8	730/38	Life	N/A	1990	No	Multivariate	Real, Integer
18	planning-relax	182	13	173/9	Computer Science	6582	2012	No	Univariate	Real
19	rice_cammeo_ osmancik	3810	7	3619/191	Biology	466,580	2019	No	Multivariate	Real
20	seismic-bumps	2584	18	2455/129	Other	4310	2013	No	Multivariate	Real
22	spambase	4601	57	4371/230	Computer Science	104,599	1999	Yes	Multivariate	Real, Integer
23	thoracic-surgery	470	16	446/24	Health and Medicine	7315	2013	No	Multivariate	Real, Integer
24	wdbc (wisconsin diagnostic breast cancer)	569	30	540/29	Health and Medicine	296,718	1995	No	Multivariate	Real

**Table 5:** The comparison of NPULUD method with DT in accuracy (%).

ID	Dataset	DT (Supervised Learning)	NPULUD (Positive Unlabeled Learning)
1	adult	86.10	86.90
2	australian-credit-approval	86.09	87.97
3	bank-marketing	91.20	91.43
4	blogger	74.00	74.00
5	blood-transfusion-service	77.81	80.88
6	breast-cancer-wisconsin	94.56	95.14
7	climate-simulation-craches	92.59	93.89
8	fertility-diagnosis	85.00	94.00
9	german-credit	70.50	75.20
10	habermans-survival	71.90	84.64
11	heart-statlog	76.67	84.44
12	hepatitis	83.87	85.81
13	ionosphere	91.45	93.45
14	kr-vs-kp	99.44	98.12
15	molecular-promotor-gene	81.13	77.36
16	parkinsons	80.51	85.64
17	pima-indians-diabetes	73.83	79.04
18	planning-relax	71.43	81.32
19	rice_cammeo_osmancik	92.49	93.75
20	seismic-bumps	93.34	97.29
21	sonar	71.15	74.04
22	spambase	92.98	92.68
23	thoracic-surgery	84.47	92.13
24	wdbc	93.15	94.55
	Average	83.99	87.24

**Table 6:** Performance of NPULUD across different ratios of positive labeled and unlabeled instances (P/U) for each dataset.

Dataset	95/5% P/U Ratio	90/10% P/U Ratio	85/15% P/U Ratio	80/20% P/U Ratio
adult	86.90	86.71	86.51	86.55
australian-credit-approval	87.97	85.94	85.94	86.52
bank-marketing	91.43	91.43	91.43	91.43
blogger	74.00	74.00	74.00	74.00
blood-transfusion-service	80.88	78.61	76.34	77.81
breast-cancer-wisconsin	95.14	95.14	95.14	95.14
climate-simulation-craches	93.89	92.59	93.33	93.33
fertility-diagnosis	94.00	92.00	92.00	92.00
german-credit	75.20	74.90	74.40	75.10
habermans-survival	84.64	82.35	81.37	82.35
heart-statlog	84.44	85.56	85.93	85.93
hepatitis	85.81	84.52	83.87	80.65
ionosphere	93.45	91.17	89.46	90.31
kr-vs-kp	98.12	97.65	98.37	98.44
molecular-promotor-gene	77.36	74.53	77.36	77.36
parkinsons	85.64	85.64	85.64	85.64
pima-indians-diabetes	79.04	76.17	77.86	76.56
planning-relax	81.32	81.32	79.67	78.02
rice_cammeo_osmancik	93.75	93.60	92.99	92.99
seismic-bumps	97.29	96.36	96.05	95.24
sonar	74.04	74.04	74.04	74.04
spambase	92.68	92.68	92.68	92.68
thoraric-surgery	92.13	91.28	90.00	89.36
wdbc	94.55	93.85	93.85	94.20
Average	87.24	86.34	86.18	86.07

**Table 7:** Comparison of the proposed method with respect to the state-of-the-art methods in terms of F-measure.

Dataset	AdaSingle-SVM [44]	AdaEnsemble-SVM [44]	AdaSingle-KNN [44]	AdaEnsemble-KNN [44]	NPULUD (Proposed)
breast-cancer-wisconsin	95.10	95.20	**96.00**	**96.00**	95.14
ionosphere	88.20	89.30	75.00	76.90	**93.45**
pima-indians-diabetes	65.50	66.00	61.10	62.40	**79.04**
sonar	67.00	68.50	62.00	63.20	**74.04**
wdbc	93.20	93.20	88.10	88.10	**94.55**
**Average**	81.80	82.44	76.44	77.32	**87.24**

## Data Availability

All datasets are publicly available in the UCI machine learning repository. (https://archive.ics.uci.edu, accessed on 2 March 2024).

## Abbreviations

The following abbreviations are used in this paper.	
AI	Artificial intelligence
AUC	Area under the curve
AUPR	Area under the precision-recall curve
DT	Decision tree
FCM	Fuzzy c-means
FCMFP	Fuzzy c-means focal point
FCM-GO	Fuzzy c-means genetic optimization
KNN	k-nearest neighbors
LR	Logistic regression
ML	Machine learning
MLP	Multi-layer perceptron
MODIS	Moderate resolution imaging spectroradiometer
MSE	Multiscale sample entropy
NB	Naive Bayes
NLCD	National land cover database
NPULUD	Neighborhood-based positive unlabeled learning using decision tree
OCSVM	One-class support vector machine
PU	Positive and unlabeled
PUL	Positive unlabeled learning
PUL-SITS	Positive and unlabeled learning of satellite image time series
RF	Random forest
RNA	Ribonucleic acid
SL	Supervised learning
SVM	Support vector machine
